# Comment on “Influence of Orotate Phosphoribosyl Transferase Levels on Tumor Prognosis and Response to Chemotherapy”

**DOI:** 10.1155/2012/263205

**Published:** 2012-10-03

**Authors:** Shailendra Kapoor

**Affiliations:** Private Practice, Mechanicsville, VA, USA

I read with great interest the recent article by Yasumatsu et al. [1]. Interestingly, recent data suggests that orotate phosphoribosyl transferase (OPRT) may significantly influence tumor prognosis and response to 5-fluorouracil therapy in a number of systemic malignancies besides head and neck carcinomas.

For instance, a close association exists between OPRT levels and prognosis in esophageal malignancies. Early stages such as stage I and stage II express higher levels of orotate phosphoribosyl transferase (OPRT) in contrast to late stages [2]. Hence, OPRT may significantly influence prognosis in esophageal carcinomas. Similarly, increased chances of metastasis following surgery for colorectal carcinomas is seen in primary tumors that show accentuated OPRT expression. An increased risk of hematogeneous metastasis is seen in colorectal tumors with elevated OPRT levels [3]. Similarly, OPRT expression enhances the antineoplastic effects of 5-fluorouracil in gastric carcinomas and markedly improves prognosis in these patients [4].

Similar, relationships are seen in pancreatic malignancies. For instance, OPRT negative tumors are associated with a lower postsurgical resection survival rates in comparison to OPRT-positive pancreatic malignancies [5]. Patients with OPRT-positive tumors who are administered chemotherapy with chemotherapeutic agents such as gemcitabine have higher survival rates in contrast to those who do not receive similar adjuvant therapy.

Similarly, there is upregulation of OPRT expression in prostate malignancies. In fact, a close association is seen between grading and tumor OPRT mRNA expression. In addition, hormone-refractory prostate cancers demonstrate a higher OPRT/dihydropyrimidine dehydrogenase (DPD) ratio in contrast to hormone-sensitive prostate cancer [6, 7]. This higher OPRT/DPD ratio explains the higher sensitivity of these tumors to 5-fluorouracil therapy. Similarly, bladder carcinomas demonstrate increased OPRT activity in contrast to normal bladder tissue. In fact, low-grade bladder carcinomas express lower levels of OPRT in contrast to high-grade bladder carcinomas. OPRT expression significantly influences the sensitivity of the tumors to 5-fluorouracil and this is an important factor in assessing prognosis in bladder carcinomas [7].

The above examples illustrate the close association between OPRT and treatment outcome and response to 5-fluorouracil in systemic malignancies and the need for further studies in this regard.

## References

[1] Yasumatsu R, Nakashima T, Komune S (2012). Overexpression of the orotate phosphoribosyl-transferase gene enhances the effect of 5-Fluorouracil in head and neck squamous cell carcinoma in vitro. *Journal of Oncology*.

[2] Takemura M, Yoshida K, Morimura K, Osugi H, Lee S, Kishida S (2010). Correlation between clinicopathological factors and enzymatic activity of orotate phosphoribosyl transferase (OPRT), dihydropyrimidine dehydrogenase (DPD) in esophageal cancer. *Gan to Kagaku Ryoho*.

[3] Akagi Y, Kinugasa T, Mizobe T, Kawahara A, Kage M, Shirouzu K (2012). Expression of dihydropyrimidine dehydrogenase, orotate phosphoribosyl transferase and thymidylate synthase in patients with primary colorectal cancer, and associations with site of first metastasis. *Anticancer Research*.

[4] Taomoto J, Yoshida K, Wada Y (2006). Overexpression of the orotate phosphoribosyl-transferase gene enhances the effect of 5-fluorouracil on gastric cancer cell lines. *Oncology*.

[5] Nio Y, Toga T, Maruyama R, Fukushima M (2007). Expression of orotate phosphoribosyl transferase in human pancreatic cancer: implication for the efficacy of uracil and tegafur-based adjuvant chemotherapy. *Oncology Reports*.

[6] Tanaka T, Kawashima H, Matsumura K (2009). Overexpression of orotate phosphoribosyl transferase in hormone-refractory prostate cancer. *Oncology Reports*.

[7] Furuse H, Hirano Y, Harada M (2004). Orotate phosphoribosyl transferase in bladder cancer. *Gan to Kagaku Ryoho*.

